# Hydrogen sulfide - cysteine cycle system enhances cadmium tolerance through alleviating cadmium-induced oxidative stress and ion toxicity in Arabidopsis roots

**DOI:** 10.1038/srep39702

**Published:** 2016-12-22

**Authors:** Honglei Jia, Xiaofeng Wang, Yanhua Dou, Dan Liu, Wantong Si, Hao Fang, Chen Zhao, Shaolin Chen, Jiejun Xi, Jisheng Li

**Affiliations:** 1 School of Environmental Science and Engineering, Shaanxi University of Science & Technology, Xi’an, Shaanxi 710021, China; 2 College of Life Sciences, Northwest A&F University, Yangling, Shaanxi 712100, China; 3 Inner Mongolia Key Laboratory of Biomass-Energy Conversion, Inner Mongolia University of Science and Technology, Baotou, Neimenggu, 014010, China; 4 Biomass Energy Center for Arid and Semi-arid Lands, Northwest A&F University, Shaanxi 712100, China; 5 Department of Grassland Science, College of Animal Science and Technology, Northwest A&F University, Shaanxi 712100, China

## Abstract

Cadmium (Cd^2+^) is a common toxic heavy metal ion. We investigated the roles of hydrogen sulfide (H_2_S) and cysteine (Cys) in plant responses to Cd^2+^ stress. The expression of H_2_S synthetic genes *LCD* and *DES1* were induced by Cd^2+^ within 3 h, and endogenous H_2_S was then rapidly released. H_2_S promoted the expression of Cys synthesis-related genes *SAT1* and *OASA1*, which led to endogenous Cys accumulation. The H_2_S and Cys cycle system was stimulated by Cd^2+^ stress, and it maintained high levels in plant cells. H_2_S inhibited the ROS burst by inducing alternative respiration capacity (AP) and antioxidase activity. H_2_S weakened Cd^2+^ toxicity by inducing the metallothionein (MTs) genes expression. Cys promoted GSH accumulation and inhibited the ROS burst, and GSH induced the expression of phytochelatin (PCs) genes, counteracting Cd^2+^ toxicity. In summary, the H_2_S and Cys cycle system played a key role in plant responses to Cd^2+^ stress. The Cd^2+^ tolerance was weakened when the cycle system was blocked in *lcddes1-1* and *oasa1* mutants. This paper is the first to describe the role of the H_2_S and Cys cycle system in Cd^2+^ stress and to explore the relevant and specificity mechanisms of H_2_S and Cys in mediating Cd^2+^ stress.

Cadmium (Cd^2+^) is a common toxic heavy metal ion in the environment. It greatly affects the growth and development of plants and is harmful to human health through the food chain^1^,^2^. Because of its carcinogenic properties and its detrimental effects on the growth of organisms, Cd^2+^ contamination of agricultural soil has become a critical concern. Preventing reduced growth and accumulation of Cd^2+^ in harvested organs of plants growing on Cd^2+^-contaminated soils has become an urgent task as it can contribute to food safety. Thus, it is important to explore plant stress defense mechanisms and to find ways to reduce the Cd^2+^ accumulation in grains.

As a heavy metal not participating in redox reactions, Cd^2+^ can easily dissolve in water and quickly be taken up by plant roots^3^,^4^. The physiological consequences of Cd^2+^ toxicity in plants are chlorosis, stunted growth, and cell death, among others^5^,^6^,^7^. At the cellular level, Cd^2+^ can alter protein structure and inhibit enzyme activity by binding to sulfhydryl and carbonyl groups and replacing essential co-factors of enzymes^7^,^8^,^9^. The overproduction of reactive oxygen species (ROS) is the primary response of plants to Cd^2+^ with negative impact on cell function^10^. Further damage can be caused by ROS-independent, secondary mechanisms. Lipid peroxidation is the most deleterious effect caused by Cd^2+^-induced ROS^4^. Malondialdehyde (MDA), one of the decomposition products of lipid peroxidation, can modify active substrates in plant cells, including nucleic acids, proteins and saccharides^11^. To become resistant to Cd^2+^ toxicity, plants have developed several strategies, such as inducing the alternative respiratory pathway^11^, activating antioxidants and glutathione (GSH)^12^, and regulating the influx and efflux of heavy metals^13^,^14^, as well as regulating the levels of heavy metal chelators, phytochelatins (PCs)^15^ and metallothioneins (MTs)^16^.

Hydrogen sulfide (H_2_S) has been considered toxic gas for many years, which inhibits cytochrome oxidase activity in animal cell^17^. Recently, it has emerged as the third endogenous gasotransmitter, following the discovery of nitric oxide and carbon monoxide^17^. In plant systems, endogenous H_2_S is generated through enzymatic pathways. Cysteine (Cys) desulfhydrases (CDes) are key enzymes involved in H_2_S generation^18^. Cys synthesis occurs via two sequential phases catalyzed by serine acetyltransferase (SAT) and O-acetylserine(thiol)lyase (OAS-TL), both of which are encoded by multigene families^19^. L-Cys desulfhydrase (LCD) is the most understood CDes in Arabidopsis; it regulates L-Cys degradation into pyruvate, ammonia and H_2_S^20^. OAS-TL regulates that H_2_S and O-acetylserine (OAS) synthetise L-Cys^20^. These physiological processes form H_2_S - Cys cycle system in cell. Recently, based on the sequence characteristics of DES1, it has been classified as an OAS-TL^21^. However, functional analysis of this enzyme revealed that DES1 has a higher affinity for L-Cys and degrades it to generate H_2_S^21^.

The alternative respiratory pathway is a unique pathway in the mitochondrial electron transport chain in higher plants that is regulated by alternative oxidase (AOX)^22^. A large body of evidence suggests that the enhanced alternative pathway could improve stress tolerance through limiting the ROS burst^22^,^23^. Recent research indicated that respiratory activity is regulated by endogenous H_2_S in *Escherichia coli*^24^. However, the relationship between H_2_S and the alternative respiratory pathway in plant responses to Cd^2+^ stress is unclear.

Sulfur is an essential element that is taken up by plants in its oxidized state, reduced to H_2_S, and first incorporated into Cys before it is used in metabolic processes. The products of sulfur metabolism, such as Cys, GSH, PCs, MTs and H_2_S, have biological functions in plant responses to heavy metal stress and oxidative stress^25^. Recently, positive effects of H_2_S in response to several types of abiotic stress in plants have been found, such as osmotic stress, salt stress, heat shock stress and heavy metal stress^26^,^27^,^28^,^29^. It has been reported that a cross-talk between H_2_S and nitric oxide is responsible for the increased Cd^2+^ tolerance in alfalfa and Bermuda grass plants^30^,^31^. In addition, H_2_S alleviates Cd^2+^ toxicity by regulating cadmium transport in *Populus euphratica* cells^32^. H_2_S is also involved in the growth and development of plants through its effects on stomatal closure and seed germination and by increasing the growth rate^33^,^34^,^35^,^36^. Cys acts as a functional precursor for many important biologic activators, such as PCs and GSH, which can enhance the tolerance of plants to heavy metal stress^25^,^37^. In addition, Cd^2+^ tolerance significantly decreases when Cys biosynthesis is blocked in *oasa1-1* and *oasa1-2* mutants^37^.

Compelling evidence has suggested that H_2_S and Cys are involved in plant tolerance to heavy metal stress. The biosynthesis of H_2_S and Cys are interrelated. H_2_S is involved in the uptake of SO_4_^2−^ and in the biosynthesis of Cys. Cys is also involved in the generation of H_2_S. Nevertheless, the relation and interaction between H_2_S and Cys are yet unknown under Cd^2+^ stress. In this study, we aimed to clarify the common and independent functions of H_2_S and Cys in Cd^2+^ stress. Additionally, we sought to demonstrate the working mechanisms of the H_2_S and Cys cycle system response to Cd^2+^ stress in Arabidopsis.

## Results

### Effect of Cd^2+^ on root elongation, MDA and EL in Arabidopsis roots

**Figure 1:**
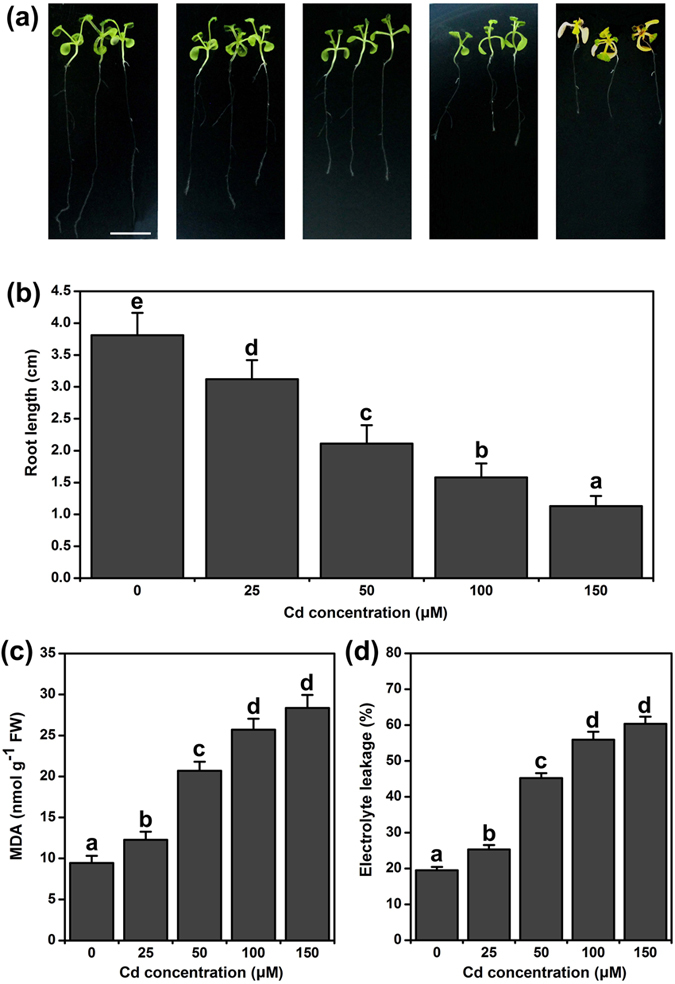
The effect of Cd^2+^ on root length, MDA and EL in Arabidopsis roots. (**a**) Phenotype of root growth in WT seedlings. Bar = 1 cm. (**b**) The root lengths of WT seedlings (n > 25). (**c**) MDA contents in WT roots stressed by various concentrations of Cd^2+^. (**d**) EL in WT roots stressed by various concentrations of Cd^2+^. 7-d-old Arabidopsis seedlings were grown on 1/2 MS agar plates supplied with 0–150 μM Cd^2+^ for 5 d, and the lengths of the primary roots, MDA contents and EL were recorded. Mean values and SE were calculated from three replicates. Within each set of experiments, bars with different letters are significantly different (*P* < 0.05, Duncan’s multiple range tests).

Arabidopsis seedlings (7-d-old) were transferred aseptically to 1/2 MS medium-containing CdCl_2_, and the lengths of the primary roots were measured 5 d later. Cd^2+^ stress led to toxicity symptoms and inhibited the elongation of Arabidopsis roots in a dose-dependent manner. As shown in Fig. 1a and b, root growth was slightly inhibited under 25 μM Cd^2+^, but root elongation was significantly inhibited under 50 to 150 μM Cd^2+^, exhibiting 53.5% to 34.9% inhibition, respectively. Malondialdehyde (MDA) and electrolyte leakage (EL) are considered as good indicators of stress-induced cell damage. Cd^2+^ stress caused lipid peroxidation and MDA accumulation. When plants were treated with 50 to 150 μM Cd^2+^, the MDA content of the roots increased by 117% to 200%, respectively (Fig. 1c). In the presence of 50 to 150 μM Cd^2+^, EL increased by 131% to 217%, respectively (Fig. 1d). The 50 to 150 μM Cd^2+^ treatment had significant effects on Arabidopsis roots. 100 and 150 μM Cd^2+^ concentrations were too violent for plant growth and 150 μM Cd^2+^ concentrations lead to the seedling etiolation in Fig. 1a. Thus, 50 μM Cd^2+^ was chosen for further study of Cd^2+^ stress.

### Effect of Cd^2+^, NaHS and Cys on the H_2_S and Cys cycle system

**Figure 2:**
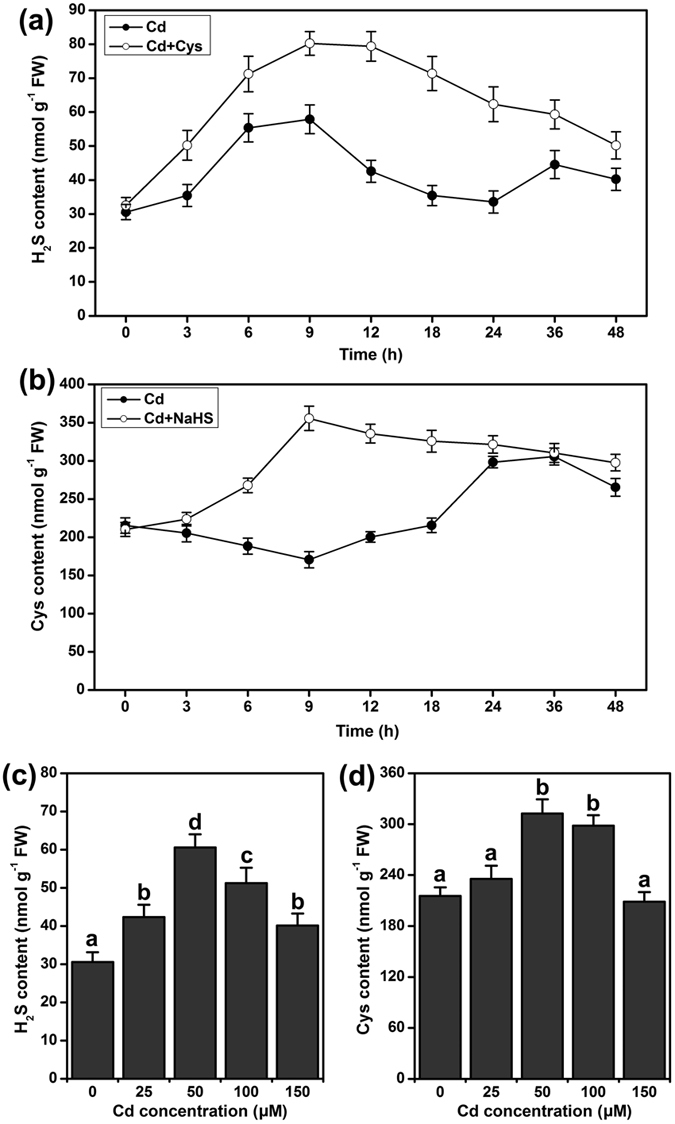
Analysis of endogenous H_2_S and Cys contents in WT roots of Arabidopsis. (**a**) Time-course of H_2_S content. (**b**) Time-course of Cys content. 7-d-old WT seedlings were treated with 50 μM Cd^2+^, 50 μM Cd^2+^ + 1 mM Cys and 50 μM Cd^2+^ + 50 μM NaHS for 0 to 48 h. (**c**) Changes of H_2_S content in various Cd^2+^ concentrations. (**d**) Changes of Cys content in various Cd^2+^ concentrations. 7-d-old WT seedlings were supplied with 0–150 μM Cd^2+^ for 5 d. Mean values and SE are calculated from three replicates. Within each set of experiments, bars with different letters are significant different (*P* < 0.05, Duncan’s multiple range tests).

To explore the working mechanisms of the H_2_S and Cys cycle system’s response to Cd^2+^ stress in Arabidopsis roots, a time-course analysis of endogenous H_2_S and Cys contents was performed. Endogenous H_2_S and Cys contents undulated along with the time of Cd^2+^ stress. H_2_S content was rapidly induced after treatment with Cd^2+^ for 3 h, reached the highest level at 9 h, and then decreased at 12 h, but had another increase at 36 h (Fig. 2a). Treatment with Cys could enhance the H_2_S level and maintain H_2_S content at a high level in Cd^2+^ stress (Fig. 2a). The Cys level slightly decreased in the initial stage under Cd^2+^ treatment, but it increased after treatment with Cd^2+^ for 12 h and maintained a high level from 24 to 36 h (Fig. 2b). Treatment with NaHS promoted Cys accumulation and a high level of Cys was maintained during Cd^2+^ stress (Fig. 2b). When plants were treated with 50 or 100 μM Cd^2+^ for a long time, both H_2_S and Cys levels were enhanced in Arabidopsis roots (Fig. 2c and d). As mentioned above, H_2_S and Cys contents were elevated by Cd^2+^ stress, and H_2_S appeared to be an important mediator in the Cd^2+^-induced increase of Cys, and the H_2_S and Cys cycle system was enhanced.

### The effects of Cd^2+^, NaHS and Cys on synthetic genes of H_2_S and Cys

**Figure 3:**
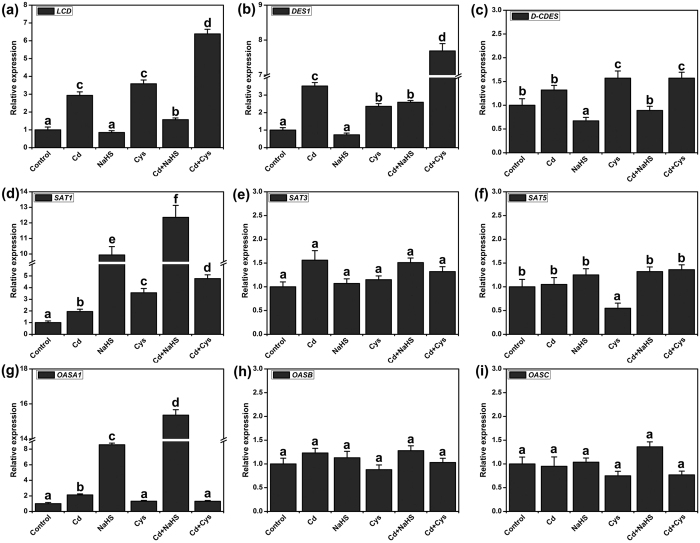
qRT-PCR analysis the synthesis genes of H_2_S and Cys in Arabidopsis WT roots. Relative expression levels were normalized with the internal standard *EF1a*. 7-d-old WT seedlings were grown on agar plates supplemented with 50 μM Cd^2+^, 50 μM NaHS, 1 mM Cys, 50 μM Cd^2+^ plus 50 μM NaHS, and 50 μM Cd^2+^ plus 1 mM Cys for 3 h, respectively. Mean values and SE were calculated from three replicates. Within each set of experiments, bars with different letters are significant different (*P* < 0.05, Duncan’s multiple range tests).

To study the direct effects of Cd^2+^, NaHS and Cys on genes regulating the synthesis of H_2_S and Cys, the Arabidopsis seedlings were exposed to various treatments for 3 h. The expressions of H_2_S synthetic genes *LCD* and *DES1* were markedly induced by Cd^2+^ and Cys (Fig. 3a and b), but the expression of *D-CDES* was not significantly affected by Cd^2+^ and Cys treatments (Fig. 3c). *SATs* and *OASs* are the important synthetic genes of Cys, but they had different responses to Cd^2+^, NaHS and Cys. The expression levels of *SAT1* and *OASA1* were slightly increased in Cd^2+^ stress, but they were markedly induced by treatment with NaHS (Fig. 3d and g). Additionally, the expression of *SAT5* was inhibited by Cys (Fig. 3f). *SAT3, OASB* and *OASC* did not respond to Cd^2+^, NaHS or Cys (Fig. 3e,h and i).

### The effects of Cd^2+^, NaHS and Cys on root elongation, MDA and EL in *lcddes1-1* and *oasa1* mutants

**Figure 4:**
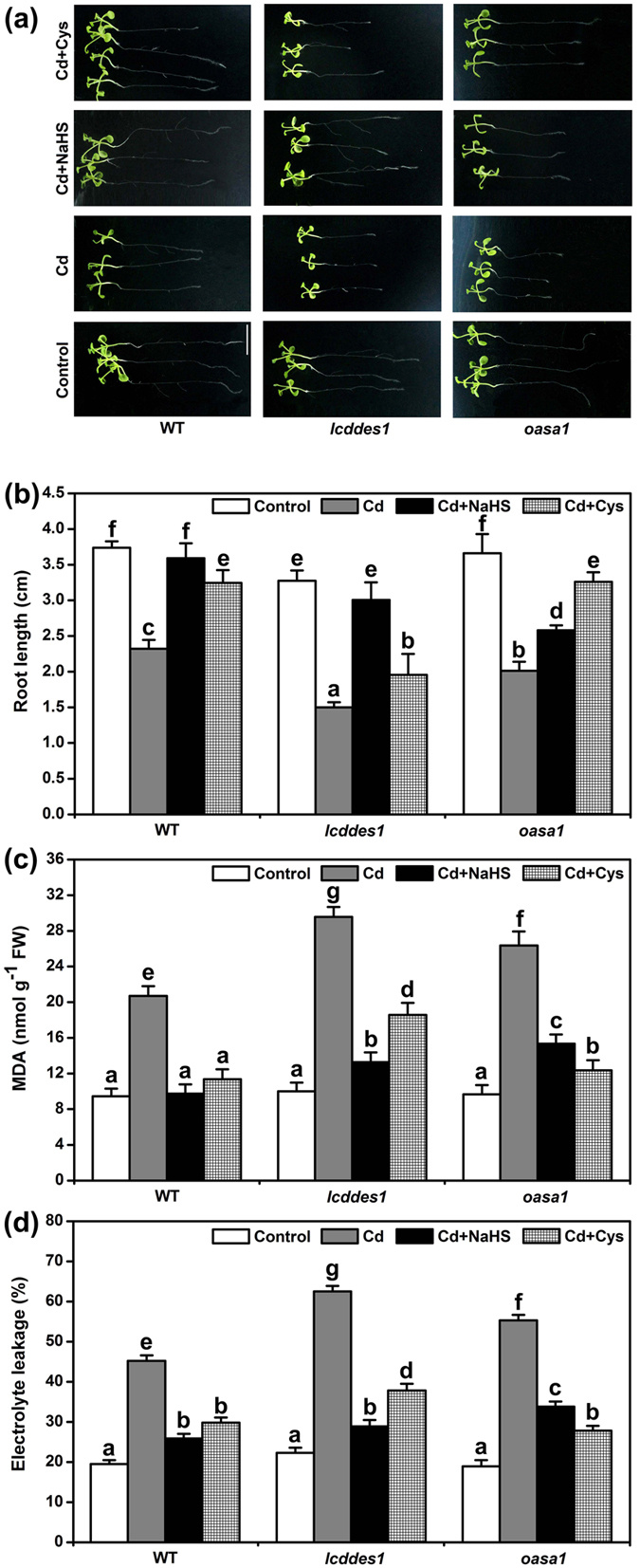
The effect of H_2_S and Cys on root length, MDA and EL in *lcddes1-1* and *oasa1* mutant plants under Cd^2+^ stress. (**a**) Phenotype of Arabidopsis root growth. Bar = 1 cm. (**b**) The root lengths of Arabidopsis seedlings (n > 25). (**c**) MDA contents in Arabidopsis roots. (**d**) EL in Arabidopsis roots. 7-d-old Arabidopsis seedlings were grown on 1/2 MS agar plates supplied with 50 μM Cd^2+^, 50 μM Cd^2+^ plus 50 μM NaHS and 50 μM Cd^2+^ plus 1 mM Cys for 5 d respectively, and the lengths of the primary roots, MDA contents and EL were recorded. Mean values and SE were calculated from three replicates. Within each set of experiments, bars with different letters are significantly different (*P* < 0.05, Duncan’s multiple range tests).

Five-day-old Arabidopsis seedlings were transferred aseptically to Cd^2+^-containing 1/2 MS medium, and the lengths of the primary roots were measured one week later. The root elongation of *lcddes1-1* mutant was shorter compared to WT root elongation under control conditions, but the root elongation of *oasa1* was the same as WT (Fig. 4a and b). The *lcddes1-1* and *oasa1* mutants were more sensitive to Cd^2+^ stress. Application of NaHS or Cys recovered the Cd^2+^-induced growth inhibition in WT. NaHS markedly recovered the effect of Cd^2+^ in *lcddes1-1*, but it only partly recovered the effect of Cd^2+^ in *oasa1* (Fig. 4a and b). On the contrary, treatment with Cys slightly recovered the effect of Cd^2+^ in *lcddes1-1*, but it significantly recovered the effect of Cd^2+^ in *oasa1* (Fig. 4a and b). Cys-mediated partial recovery the root length may be due to an independent physiological action of Cys in *lcddes1-1* because H_2_S production was blocked in the double mutant. NaHS or Cys treatment markedly decreased the EL level and the content of MDA under Cd^2+^ stress in WT (Fig. 4c and d). NaHS strongly reduced the MDA content and the EL in *lcddes1-1* and *oasa1* (Fig. 4c and d). Cys prevented the effects of Cd^2+^ on the MDA content and EL in *oasa1* but partly weakened the effects of Cd^2+^ on the MDA content and EL in *lcddes1-1* (Fig. 4c and d).

### The effects of Cd^2+^, NaHS and Cys on the alternative respiratory pathway

**Figure 5:**
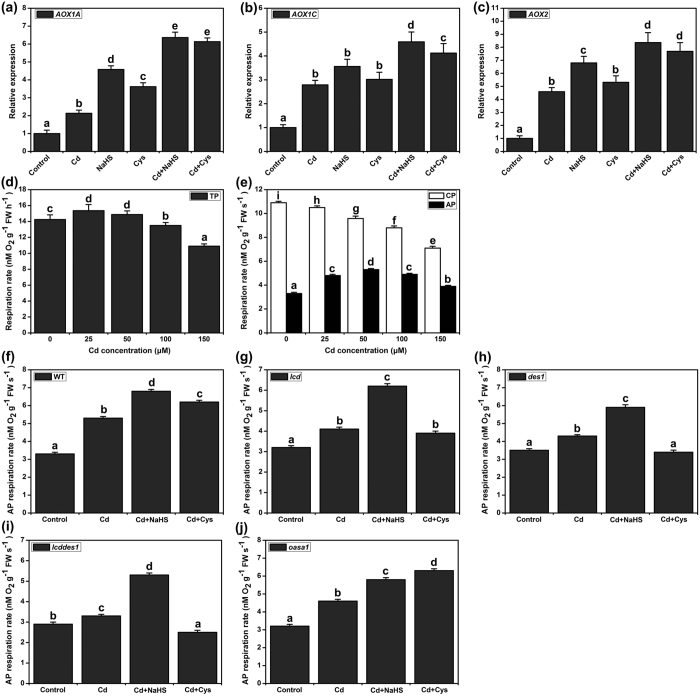
Effect of H_2_S and Cys on the expression of AOX genes and the activity of TP, CP and AP in Cd^2+^ stress. (**a**–**c**) The expression of *AOX1A, AOX1C* and *AOX2*. 7-d-old WT seedlings were grown on 1/2 MS agar plates supplied with 50 μM Cd^2+^, 50 μM NaHS, 1 mM Cys, 50 μM Cd^2+^ plus 50 μM NaHS, and 50 μM Cd^2+^ plus 1 mM Cys for 3 h, respectively. (**d**,**e**) Changes in TP, CP and AP activity in various Cd^2+^ concentrations for 5 d in WT. (**f**–**l**) AP activity in WT and mutants. 7-d-old Arabidopsis seedlings were grown on 1/2 MS agar plates treated with 50 μM Cd^2+^, 50 μM Cd^2+^ plus 50 μM NaHS and 50 μM Cd^2+^ plus 1 mM Cys for 5 d. Mean values and SE were calculated from three replicates. Within each set of experiments, bars with different letters are significantly different (*P* < 0.05, Duncan’s multiple range tests).

The alternative respiratory pathway plays an important role in plant stress resistance by limiting the ROS burst^38^. In this study, we sought to elucidate the roles of NaHS and Cys in the alternative respiratory pathway under Cd^2+^ stress. In general, the alternative pathway operates at a low level under normal conditions, but it can be significantly induced when plants are stimulated by various environmental stresses^23^. We first checked the expression of AOX genes after Cd^2+^, NaHS and Cys treatments for 3 h. The expression of *AOX1A, AOX1C* and *AOX2* were increased in Cd^2+^ stress (Fig. 5a,b and c). Interestingly, NaHS and Cys treatments also markedly enhanced the expression levels of *AOX1A, AOX1C* and *AOX2* in both control and Cd^2+^ stress conditions (Fig. 5a,b and c). Furthermore, the total respiration capacity (TP), cytochrome respiration capacity (CP) and alternative respiration capacity (AP) were analyzed in WT and mutant plants. TP was slightly enhanced by 25 or 50 μM Cd^2+^, but it was inhibited by 100 or 150 μM Cd^2+^ in WT (Fig. 5d). Under Cd^2+^ stress, CP was inhibited in a dose-dependent manner; however, AP was increased under Cd^2+^ stress, and AP achieved its maximum induction with the 50 μM Cd^2+^ treatment (Fig. 5e). Similar to the pattern of expression of the AOX genes in response to NaHS or Cys treatments under Cd^2+^ stress, AP was increased by NaHS or Cys under Cd^2+^ stress (Fig. 5f). However, it was different in the mutant plants. Under Cd^2+^ stress, the effects of Cys on AP were not observed in *lcd* and *des1-1*, and they were especially decreased in *lcddes1-1* (Fig. 5g,h and i). In *oasa1*, the effects of NaHS and Cys were the same as in WT under Cd^2+^ stress (Fig. 5g).

### The effects of Cd^2+^, NaHS and Cys on antioxidant enzyme activity and GSH level, and the relationship among AP, antioxidant enzyme activity, and GSH level in Cd^2+^ stress

**Figure 6:**
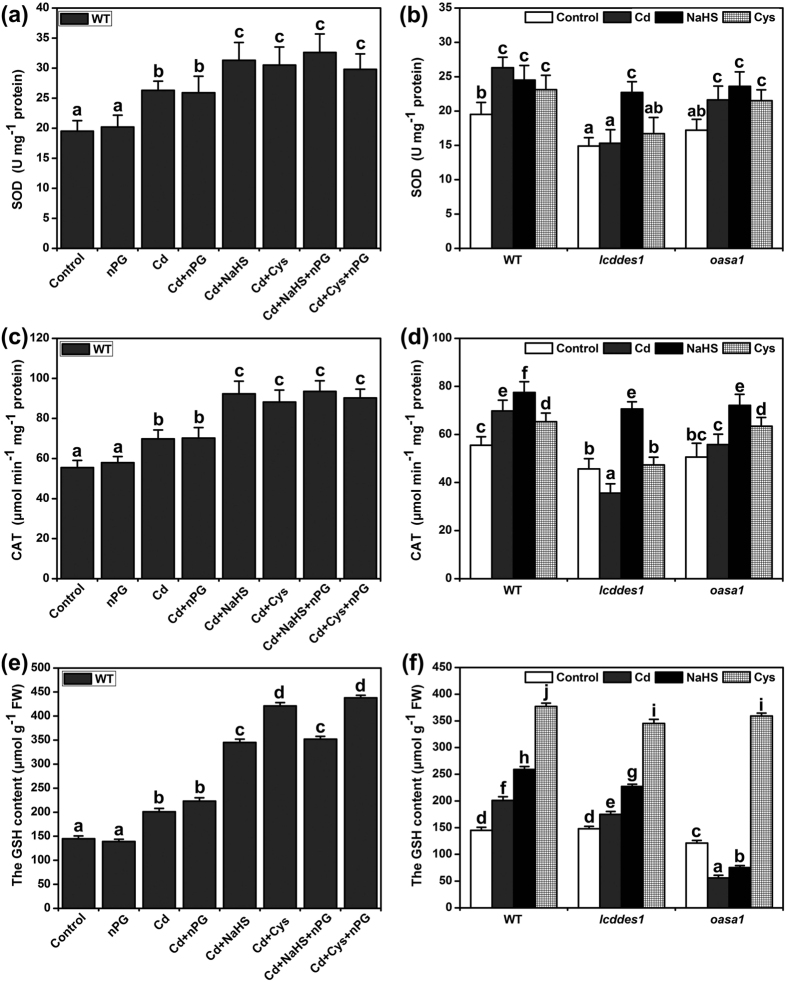
Effect of H_2_S and Cys on antioxidant enzymes activity and GSH level in Cd^2+^ stress. 7-d-old Arabidopsis seedlings were grown on 1/2 MS agar plates supplemented with 50 μM Cd^2+^, 50 μM NaHS, 1 mM Cys, and 200 μM nPG for 6 h, and SOD activity (**a**,**b**), CAT activity (**c**,**d**) and GSH content (**e**,**f**) were recorded. Mean values and SE were calculated from three replicates. Within each set of experiments, bars with different letters are significantly different (*P* < 0.05, Duncan’s multiple range tests).

Antioxidant enzymes depress the level of ROS. A previous study showed that H_2_S could enhance antioxidase activity in rice^39^. In addition, many studies suggested that AOX was important in maintaining the homeostasis of the redox state^22^,^38^. Therefore, the effects of Cd^2+^, NaHS, Cys and AP on antioxidant enzyme activity were analyzed. As shown in Fig. 6a and c, after 12 h of 50 μM Cd^2+^ treatment, the activities of SOD and CAT in plants were significantly higher than in the control plants in WT. NaHS or Cys treatments could enhance the antioxidase activity under unstressed conditions (Fig. 6b and d), and this enhancement was further strengthened under Cd^2+^ stress in WT (Fig. 6a and c). However, treatment with n-propyl gallate (nPG) had no significant effect on the antioxidase activity of the plants either under Cd^2+^ stress or under unstressed conditions. Furthermore, nPG did not affect the elevated antioxidase activity of the NaHS- and Cys-treated plants under Cd^2+^ stress (Fig. 6a and c). The effects of Cd^2+^ and Cys were altered in *lcddes1-1*; treatment with Cd^2+^ or Cys did not enhance the antioxidase activity in *lcddes1-1* (Fig. 6b and d). Additionally, the effects of Cd^2+^ on the SOD activity were also weakened, and CAT activity was negligible in *oasa1* (Fig. 6d). Contrarily, treatment with NaHS still enhanced the antioxidase activity in mutant plants (Fig. 6b and d).

GSH is the product of sulfur metabolism, and it has positive biological functions in plant responses to heavy metal stress and oxidative stress^25^. As shown in Fig. 6e, the GSH content was increased in Cd^2+^ stress. NaHS and Cys also enhanced the GSH level in WT (Fig. 6e and f). Specially, Cys had a significant promoting effect on GSH content. The *oasa1* mutant did not respond to Cd^2+^ and NaHS, and even had a reduced GSH level, but Cys still increased the GSH content in *oasa1* (Fig. 6f). Additionally, the effect of Cd^2+^, NaHS and Cys on the GSH content in *lcddes1-1* was the same as WT plants (Fig. 6f).

### Effect of NaHS and Cys on ROS, and the relationship between AP and ROS in Cd^2+^ stress

**Figure 7:**
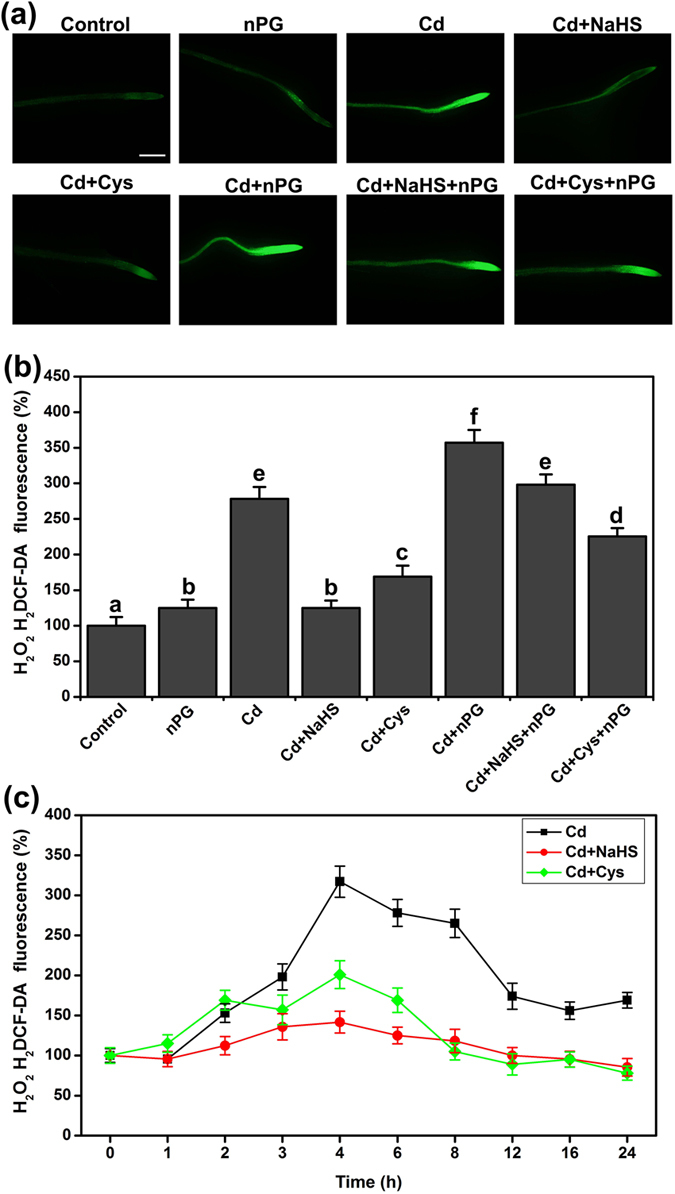
The changes of the endogenous H_2_O_2_ level in Arabidopsis root. (**a**) H_2_O_2_ H_2_DCF-DA fluorescence in WT roots (bar = 100 μm). (**b**) Quantification of H_2_O_2_ H_2_DCF-DA fluorescence intensity. (**c**) Time-course of H_2_O_2_ level. 7-d-old Arabidopsis seedlings were grown on 1/2 MS agar plates supplemented with 50 μM Cd^2+^, 50 μM NaHS, 1 mM Cys, and 200 μM nPG for 6 h (**a**,**b**), 0–24 h (**c**). H_2_DCF-DA fluorescence intensity data represents mean grey values. SE was calculated from measurements of at least five roots for each treatment, and the experiments were repeated three times. Within each set of experiments, bars with different letters are significantly different (*P* < 0.05, Duncan’s multiple range tests).

To estimate the potential role of the H_2_S and Cys cycle in ROS homeostasis, we visualized the production of H_2_O_2_ in the roots under Cd^2+^ stress. Over-accumulation of H_2_O_2_ was visualized by fluorescence labeling in the roots subjected to Cd^2+^ stress (Fig. 7a). Conversely, NaHS or Cys treatment considerably diminished the accumulation of H_2_O_2_ in Cd^2+^ stress (Fig. 7a and b). Additionally, inhibiting the alternative respiratory pathway with nPG caused an over-accumulation of H_2_O_2_ under Cd^2+^ stress. The effects of NaHS and Cys were partly averted and slightly weakened by nPG in Cd^2+^ stress, respectively (Fig. 7a and b). As shown in the time-course of H_2_O_2_, the ROS burst occurred during the early phase of Cd^2+^ stress. Then, high levels of ROS were maintained from 4 to 8 h and declined after 12 h. H_2_S supplementation could maintain H_2_O_2_ at a low level during Cd^2+^ stress. Treatment with Cys did not alter the burst of H_2_O_2_ in the early phase, but it prevented the over-accumulation of H_2_O_2_ after 6 h (Fig. 7c).

### Effect of NaHS and Cys on Cd^2+^ accumulation

**Figure 8:**
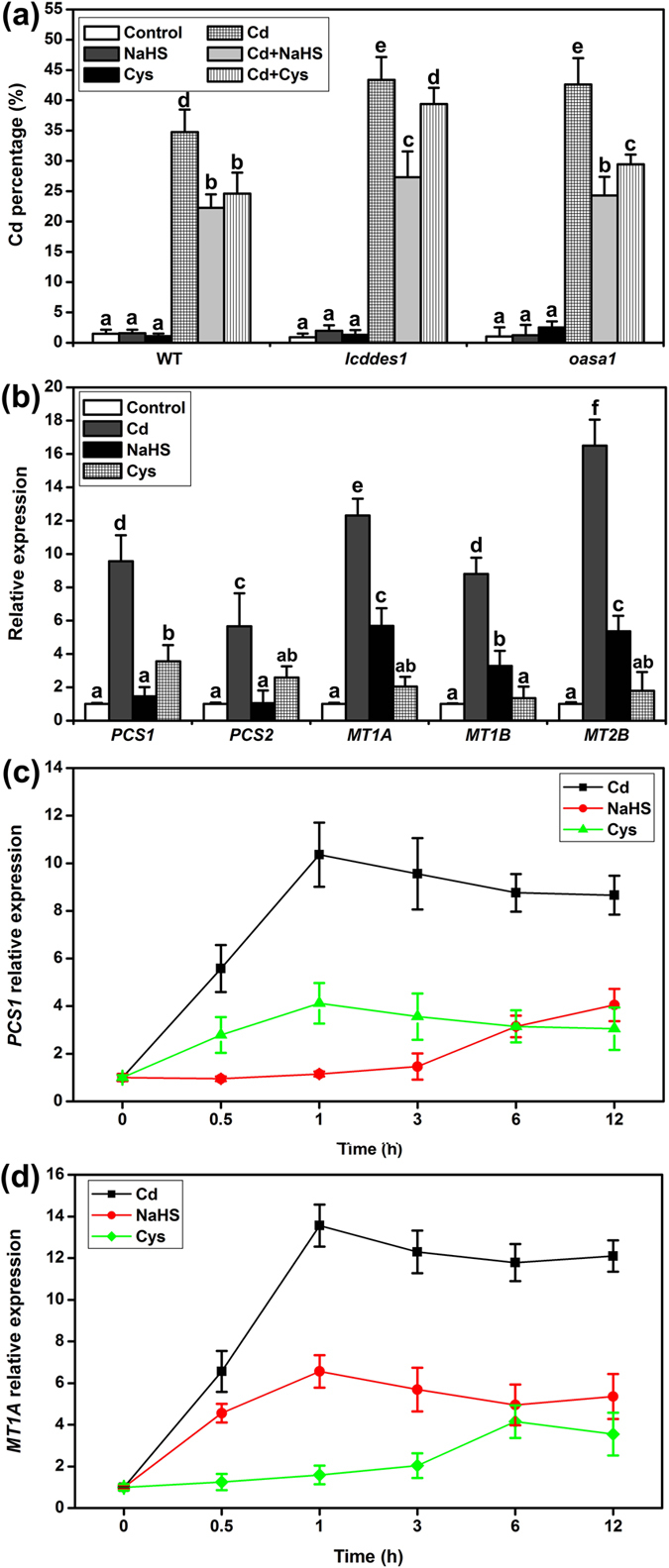
Effect of NaHS and Cys on Cd^2+^ percentage and qRT-PCR analysis the expression of heavy metal chelators genes in Arabidopsis roots. (**a**) Cd^2+^ accumulation in Arabidopsis roots. (**b**) The expression of *PCS1, PCS2, MT1A, MT1B* and *MT2B* in WT. (**c**) Time-course of *PCS1* gene expression. (**d**) Time-course of *MT1A* gene expression. 7-d-old Arabidopsis seedlings were grown on 1/2 MS agar plates supplied with 50 μM Cd^2+^, 50 μM NaHS and 1 mM Cys for 5 d (**a**), 3 h (**b**), 0–12 (**c**,**d**). Mean values and SE were calculated from three replicates. Within each set of experiments, bars with different letters are significantly different (*P* < 0.05, Duncan’s multiple range tests).

The role of H_2_S and Cys in Cd^2+^ homeostasis was investigated by measuring the percentage of Cd^2+^ in the root. The results in Fig. 8a show that Cd^2+^ accumulation increased in roots under Cd^2+^ stress in WT and in the mutants, but the mutants accumulated more Cd^2+^ than the WT. NaHS or Cys supplementation had inhibitory effects on Cd^2+^ uptake and accumulation in WT and *oasa1*. Nevertheless, *lcddes1-1* did not respond to the effect of NaHS under Cd^2+^ stress, but the effect of Cys on Cd^2+^ uptake and accumulation was only partially reduced in *lcddes1-1*.

### Effect of NaHS and Cys on the expression of heavy metal chelator genes

When plants were treated with Cd^2+^ for 3 h, the expression of the heavy metal chelator genes *PCS1, PCS2, MT1A, MT1B* and *MT2B* was significantly up-regulated in WT. Cys supplementation promoted the expression of *PCS1* and *PCS2*, and NaHS promoted the expression of *MT1A, MT1B* and *MT2B* (Fig. 8b). To further study the effect of the H_2_S and Cys cycle system on the heavy metal chelator genes, the time-course of *PCS1* and *MT1A* gene expression was investigated. Cd^2+^ was found to up-regulate the expression of *PCS1* and *MT1A* genes at 0.5 h, which then remained at a high expression level. Cys enhanced the expression of the *PCS1* gene at 0.5 h, which reached a maximum by 1 h, but NaHS enhanced the expression of the *PCS1* gene at 6 to 12 h. The expression of *MT1A* was different from *PCS1*. After 3 h of Cys supplementation, the expression of *MT1A* started to increase, reaching a maximum at 6 h, and NaHS enhanced the expression of *MT1A* gene at 0.5 h (Fig. 8c and d).

## Discussion

The root is the primary organ that plants deploy to accumulate most of the heavy metals to which they are exposed^40^,^41^. Sulfur metabolism is required for the growth and development of plants, and the production of sulfur metabolites also plays a critical role in plant responses to heavy metal-induced stress^25^. H_2_S and Cys are important sulfur metabolism products that participate in suppressing heavy metal stress in plants^40^. In previous reports, H_2_S and Cys were always studied separately in plant responses to abiotic stress^39^,^42^. Recently, H_2_S and H_2_S-induced Cys accumulation were reported to be critical in imparting Cr^6+^ tolerance in Arabidopsis^43^. Therefore, the H_2_S and Cys cycle is an important system for regulating H_2_S and Cys functions in heavy metal stress. In this study, we used the *lcddes1-1* and *oasa1* Arabidopsis mutants to block the H_2_S and Cys cycle system. Then, we intensively researched the relevant and specificity roles of H_2_S and Cys in Cd^2+^ stress. Our results indicated that Cd^2+^ can rapidly accumulate in Arabidopsis roots and inhibit the primary root growth in a Cd^2+^ concentration-dependent manner (Figs 1a and 7a), suggesting that Cd^2+^ is easily absorbed and highly toxic. We observed that endogenous H_2_S and Cys levels undulate from 3 to 48 h under Cd^2+^ stress (Fig. 2). However, the endogenous patterns of change were different for H_2_S and Cys levels. Endogenous H_2_S was first induced by Cd^2+^ stress, and then Cys levels increased. On this account, we suppose that H_2_S is produced rapidly under Cd^2+^ stress and that it acts as second messenger to activate the synthesis of Cys, implying that Cd^2+^ stress could be the direct cause of endogenous H_2_S release but that Cys accumulation is a secondary effect of Cd^2+^ stress. Data for the expression of H_2_S and Cys synthetic genes supports this hypothesis. The expression of H_2_S synthetic genes was directly induced by Cd^2+^, and then, exogenous H_2_S supplementation induced the upregulation of Cys synthetic-related genes (Fig. 3). Additionally, exogenous H_2_S or Cys supplementation during Cd^2+^ stress could rapidly induce mutual endogenous levels of the other contents in Cd^2+^ stress. These results suggested that the H_2_S - Cys cycle system was triggered by Cd^2+^ and that H_2_S and Cys could promote the production of each other, forming a cycle of activation. Finally, treatment with Cd^2+^ for 5 d, H_2_S and Cys contents increased significantly in Arabidopsis roots.

The expression levels of the Cys synthesis-related genes *OASA1* and *SAT1* were up-regulated significantly by H_2_S treatment, and the H_2_S synthesis genes *LCD* and *DES1* were up-regulated significantly by Cys treatment (Fig. 3). *OASA1* directly regulated Cys synthesis, and *LCD* and *DES1* directly regulated H_2_S synthesis; thus, *lcd, des1-1, lcddes1-1* and *oasa1* were used to study the H_2_S and Cys cycle system in Cd^2+^ stress. The Cd^2+^-induced root shortening and increases in MAD and EL were markedly enhanced in mutant plants, suggesting that the Cd^2+^ resistance was weakened when the H_2_S and Cys cycle was blocked. Exogenous H_2_S or Cys supplementation only partly restored the root growth, MAD and EL levels, suggesting that H_2_S or Cys alone could not replace the function of the H_2_S and Cys cycle in plant cells. Additionally, the H_2_S and Cys system is also important for stress caused by other heavy metals, such as Cr^6+^; it was reported that NaHS treatment increases the expression levels of the Cys synthesis-related genes^43^. However, different heavy metal stress condition lead to the difference genes expression include MTs genes^43^, thus Cd^2+^ and Cr^6+^ condition also could lead to the difference MTs genes expression. The details regarding the mechanism of H_2_S in heavy metal resistance requires further study.

Excessive Cd^2+^ can induce the production of ROS, which is highly toxic to biomembranes, nucleic acids and proteins^11^. The alternative respiratory pathway plays an important role in stress conditions by repressing the production of ROS^22^,^23^,^42^. Our study also found that the CP and AP activities were altered by Cd^2+^ stress (Fig. 5e). Plant signaling molecules, such as nitric oxide, can regulate the alternative respiratory pathway in stress conditions^44^. Whether an H_2_S signal or Cys could affect AP activity was not previously known; our analysis found that exogenous H_2_S or Cys supplementation could further induce the activity of AP in Cd^2+^ stress. However, in H_2_S synthesis mutants, the effect of Cys was negligible, and in Cys synthesis mutants, the effect of H_2_S was not altered. These data imply that H_2_S is a direct trigger of AP activity and that Cys might play an indirect role in Cd^2+^ stress. The expression of AOX genes was also induced by H_2_S within 3 h, but not by Cys.

Antioxidases are also one of the central elements in maintaining ROS levels in plant cells^45^. We investigated the connection between the alternative respiratory pathway and antioxidases, but we found that the activities of SOD and CAT were not altered when the alternative respiratory pathway was inhibited by nPG (Fig. 6a and c), suggesting that the alternative respiratory pathway and antioxidases have independent functions in response to Cd^2+^ stress. The activities of SOD and CAT were induced by Cd^2+^ and increased Cd^2+^ resistance (Fig. 6). H_2_S or Cys biosynthesis was necessary for the increase in SOD and CAT activities in response to Cd^2+^ stress because Cd^2+^-induced activities of SOD and CAT were weakened in H_2_S and Cys synthesis mutants. We further studied the relationship of H_2_S and Cys in this physiological process. H_2_S supplementation could remedy the deficiency of Cys biosynthesis and increase the activities of SOD and CAT in *oasa1* mutants, but Cys supplementation could not. These data suggest that the activities of SOD and CAT are directly regulated by H_2_S and that Cys indirectly affects the activities of SOD and CAT by promoting the generation of H_2_S.

GSH performs numerous physiological functions in the plant response to heavy metal stress^46^. Cys is a precursor of GSH, which stores and transports GSH via the γ-glutamyl cycle^47^. In this study, supplementation with exogenous H_2_S or Cys strengthened Cd^2+^-mediated GSH elevation in WT plants (Fig. 6f). It is interesting that the effects of Cd^2+^ and H_2_S were reversed in *oasa1*, but the effects of Cd^2+^ and H_2_S were not altered in *lcddes1-1* (Fig. 6f). These results suggest that Cys is a direct regulatory factor of GSH, and H_2_S affects GSH levels indirectly. Additionally, the GSH content was not altered by nPG (Fig. 6e), suggesting that the alternative respiratory pathway and GSH are not related in their responses to Cd^2+^ stress.

Cd^2+^ enrichment was also observed in this study (Fig. 8a). Inhibiting Cd^2+^ uptake and enhancing Cd^2+^ efflux are the main defense strategies that plant cells use to prevent Cd^2+^ toxicity. Exogenous H_2_S or Cys supplementation effectively inhibited the accumulation of Cd^2+^ (Fig. 8a). When endogenous H_2_S or Cys synthesis was blocked, Cd^2+^ over-accumulation occurred (Fig. 8a), suggesting that the H_2_S and Cys cycle system is important for inhibiting Cd^2+^ uptake or enhancing Cd^2+^ efflux. Additionally, the effect of Cys was partly inhibited in the *lcddes1-1* mutant, implying that the role of H_2_S in the H_2_S and Cys cycle might be to directly regulate Cd^2+^ uptake or efflux.

The generation of chelators is also an effective pathway in plant cells for avoiding Cd^2+^ toxicity. *PCS1, PCS2, MT1A, MT1B* and *MT2B* are mainly expressed in roots and regulate PCs and MTs synthesis; the expression of these chelators is generally induced by numerous heavy metal ions^42^,^43^,^48^. Interestingly, the expression of *PCS1* and *PCS2* was found to be induced by Cys in a very short time, and the expression of *MT1A, MT1B* and *MT2B* was induced by H_2_S (Fig. 8b). However, only long-term supplementation of Cys or H_2_S induced the expression of *PCS1* and *MT1A* (Fig. 8c,d). These data suggest that the generation of chelators can be regulated differently in plant cells. Cys and H_2_S played different roles in the physiological process, but when combined Cys and H_2_S mutually promoted the expression of chelator synthesis genes to a level higher than when they were used as separate supplements.

**Figure 9:**
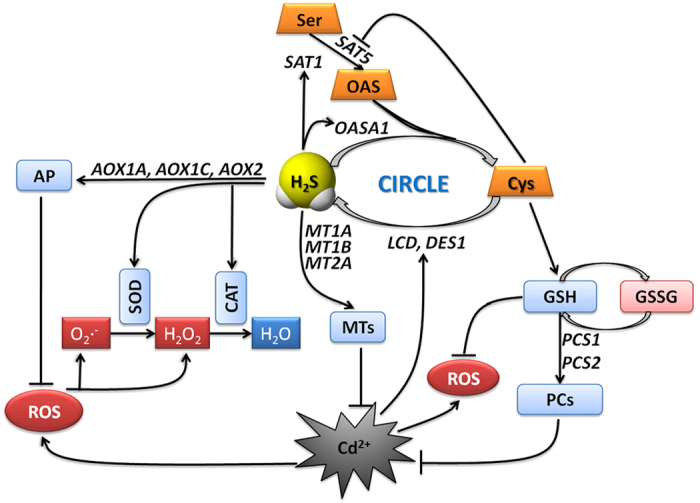
A diagram representing Cd^2+^-induced toxicity and protective mechanism of the H_2_S and Cys cycle system in Arabidopsis roots. Arrows indicate enhanced effects and hyphens indicate suppressed effects.

Based on the data described above, a signal pathway model was developed and is depicted in Fig. 9. It shows the specific roles of H_2_S and Cys in regulating plant responses to Cd^2+^ stress and their interaction. H_2_S is activated much earlier than Cys in plant responses to Cd^2+^ stress, acting as a secondary messenger to increase Cys accumulation by regulating the transcription levels of *SAT1* and *OASA1*. In addition, the production of H_2_S might deplete the endogenous Cys pool, which might subsequently increase the expression of *SAT1* or *OASA1*. Furthermore, once the H_2_S and Cys cycle is initiated, it works to maintain elevated H_2_S and Cys levels. H_2_S inhibits the ROS burst by promoting CP and antioxidase activities, and it weakens Cd^2+^ ion toxicity by inducing the gene expression of MTs. Cys acts as a precursor of GSH to promote GSH accumulation, which then contributes to inhibiting the ROS burst. GSH also induces genes expression of PCs, leading to raised PC activity, which counteracts Cd^2+^ ion toxicity. In sum, the H_2_S and Cys cycle system is a key regulator of the response to Cd^2+^ stress in plants that acts to induce and maintain levels of bioactive molecules (H_2_S, Cys, GSH, PCs, and MTs) that improve plant resistance to Cd^2+^ stress.

## Materials and Methods

### Plant material and chemical treatments

This study was carried out on *Arabidopsis thaliana,* including wild ecotypes Columbia (Col-0) and the *lcd* (SALK_082099), *des1-1* (SALK_103855), *lcddes1-1* and *oasa1* (SALK_074242c) mutants. Seeds were surface sterilized with 70% ethanol for 30 s and 15% sodium hypochlorite for 15 min and were washed at least five times with sterilized water before sowing on solid 1/2 Murashige and Skoog (MS) medium (pH 5.7), which contained 1% (w/v) sucrose, and 0.8% (w/v) agar. After that, the seeds were vernalized for 48 h at 4 °C. Then, the seedlings were grown in a growth room, which had the temperature set at 22 °C and a 14/10 h light/dark photoperiod under a photon flux density of 120 mmol m^−2^s^−1^. The Arabidopsis plants used throughout this work were grown routinely in a growth chamber under 50–60% humidity.

Following 7 d growth, Arabidopsis seedlings were transferred to the following mediums: (1) 1/2 MS agar medium, (2) 1/2 MS agar medium containing 25–150 μM CdCl_2_, 50 μM sodium hydrosulfide (NaHS), 1 mM Cys, or 200 μM n-propyl gallate (nPG), respectively. The H_2_S donors NaHS, Cys and nPG were purchased from Sigma (USA).

### Root elongation assays

Seven-day-old Arabidopsis seedlings grown on the vertical 1/2 MS agar plates were transferred to the 1/2 MS agar medium containing various chemicals for the different treatments. Root elongation was measured after 5 d of various treatments. All experiments were repeated at least three times, with photographs collected at 7 d from one representative experiment being shown. The root length was measured with ImageJ.

### Electrolyte leakage assay

Measurement of ion leakage was determined according to Sairam and Srivastava (2002) with some modifications^43^. The 7-d-old Arabidopsis seedlings were treated for 5 d on the 1/2 MS agar medium containing different chemicals. Following the treatments, the roots were collected and washed in deionized water three times to remove surface-adhered electrolytes. Then, they were immersed in 10 ml deionized water for 3 h at 25 °C in test tubes. After the incubation, the conductivity in the bathing solution was determined (*C*_*1*_), and the conductivity of deionized water was also determined (*C*_*0*_). The samples were heated in boiling water for 1 h before the total conductivity was measured in the bathing solution (*C*_*2*_). Relative ion leakage was expressed as a percentage of the total conductivity after heating in boiling water [relative ion leakage = (*C*_*1*_ − *C*_*0*_)/(*C*_*2*_ − *C*_*0*_) × 100].

### MDA and GSH content assays

The chemical treatments were the same as the measurements of ion leakage. Following the treatments^49^, Arabidopsis roots were collected. Lipid peroxidation of the roots was measured by estimating the MDA content according to the method of Heath and Packer. The GSH content was measured based on a previously described method^49^.

### Measurement of H_2_S content

H_2_S quantification was performed as described by Nashef *et al*.^50^. The chemical treatments were the same as the methods of ion leakage. Following the treatments, the seedling roots were collected with liquid nitrogen and ground into fine powder with mortar and pestle; 0.3 g of frozen tissue was homogenized in 1 ml 100 mM potassium phosphate buffer (pH = 7), which contained 10 mM ethylenediaminetetra-acetic acid (EDTA). The homogenates were centrifuged at 15,000 × *g* for 20 min at 4 °C, and 100 μl of supernatant was used for the quantification of H_2_S in an assay mixture containing 1,880 μl extraction buffer and 20 μl of 20 mM 5,5′-dithiobis (2-nitrobenzoic acid), for a total volume of 2 ml. The assay mixture was incubated at room temperature for 2 min, and the absorbance was read at 412 nm. H_2_S was quantified based on a standard curve of known concentrations of NaHS.

### Measurement of the Cys content

The chemical treatments were the same as the measurements of ion leakage. Following the treatments, Arabidopsis roots were collected. Cys can react specifically with acid ninhydrin, and the product was extracted by methylbenzene, which has a maximum absorbance at 560 nm. The reaction is highly sensitive for Cys determination. Thus, the Cys content could be determined as described previously^51^.

### RNA isolation and qRT-PCR

Seven-day-old Arabidopsis seedlings were transferred to the 1/2 MS agar medium containing different chemicals and treated for 0–12 h. Following the treatments, roots of Arabidopsis were harvested to extract total RNA for real-time polymerase chain reaction (RT-PCR). Total RNA was extracted using an RNAprep pure plant kit (Tiangen, Beijing) and was treated with RNase-free DNase reagent (RNase-free DNase kit, Tiangen). The total RNA was reverse-transcribed into first-strand cDNA using PrimeScript™ Reverse Transcriptase (Takara, Japan) and Oligo (dT)_15_ primer (Takara) following the manufacturer’s instructions. The samples were amplified using SYBR Green I (SYBR^®^ Premix Ex Taq™ Kit, Takara). The housekeeping gene EF1A was used as an internal control. The thermal cycle used was as follows: 95 °C for 10 s, and 40 cycles of 95 °C for 5 s and 59 °C for 25 s. This was followed by 80 cycles of 10 s during the time elapsed at 55–95 °C. The PCR amplifications for each gene were performed in triplicate. The results were analyzed by Rotor-Gene Real-Time Analysis Software 6.1 (Build 81).

### Extraction and assay of antioxidant enzymes

Seven-day-old Arabidopsis seedlings were transferred to the 1/2 MS agar medium containing different chemicals and treated for 6 h. Following the treatments, Arabidopsis roots were collected and enzymes extracted according to the method of Mostofa *et al*.^51^. Activities of antioxidase and glyoxalase were determined by the standard methods reported in Mostofa and Fujita^52^ for SOD (EC 1.15.1.1) and CAT (EC 1.11.1.6). The protein standard was bovine serum albumin (BSA), which was employed to determine the protein content.

### Determination of H_2_O_2_ contents

H_2_O_2_ was visualized using the specific H_2_O_2_ fluorescent probe dichlorofluorescein diacetate (H_2_DCF-DA) according to the method described by Maffei *et al*.^53^. Seven-day-old Arabidopsis seedlings were transferred to the 1/2 MS agar medium containing different chemicals and treated for 0–24 h. Following the treatments, Arabidopsis seedlings were incubated in the reaction buffer containing 10 mM 4-(2-hydroxyethyl)-1-piperazine-ethanesulfonic acid (HEPES)-NaOH (pH 7.5) and 10 μM H2DCF-DA for 15 min at 25 °C. Thereafter, the roots were washed three times with the HEPES-NaOH buffer (pH 7.4) prior to visualization using a laser confocal scanning microscope (Leica SM IRBE Multisync FE 1250). Excitation was at 480 nm and emission was at 520 nm. Images were processed and analyzed using the Leica Tcs SP2 software.

### Statistical analysis

Each experiment was repeated at least three times and with three replications each time. Values were expressed as the mean ± SE. Experiments that required an analysis of variance were analyzed using SPSS 17.0 for one-way analysis of variance (ANOVA) followed by Dunnett’s post hoc multiple comparisons. The confidence coefficient was set at 0.05.

## Additional Information

**How to cite this article:** Jia, H. *et al*. Hydrogen sulfide - cysteine cycle system enhances cadmium tolerance through alleviating cadmium-induced oxidative stress and ion toxicity in Arabidopsis roots. *Sci. Rep.* **6**, 39702; doi: 10.1038/srep39702 (2016).

**Publisher's note:** Springer Nature remains neutral with regard to jurisdictional claims in published maps and institutional affiliations.

## Supplementary Material

Supplementary Dataset 1

## References

[b1] Bolan N. S. . Chapter Four—Cadmium Contamination and Its Risk Management in Rice. Ecosystems Adv. Agron. 119, 183–273 (2013).

[b2] Sun H. . Association of cadmium in urine and blood with age in a general population with low environmental exposure. Chemosphere. 156, 392–397 (2016). 27186688 10.1016/j.chemosphere.2016.05.013

[b3] Kan Q. . Nitrate reductase-mediated NO production enhances Cd accumulation in Panax notoginseng roots by affecting root cell wall properties. J. Plant Physiol. 193, 64–70 (2016). 26956919 10.1016/j.jplph.2016.01.017

[b4] Sandalio L. M., Dalurzo H. C., Gómez M., Romero-Puertas M. C. & del Río L. A. Cadmium-induced changes in the growth and oxidative metabolism of pea plants. J. Exp. Bot. 52, 2115–2126 (2001). 11604450 10.1093/jexbot/52.364.2115

[b5] Ortega-Villasante C., Rellán-Álvarez R., Del Campo F. F., Carpena-Ruiz R. O. & Hernández L. E. Cellular damage induced by cadmium and mercury in Medicago sativa. J. Exp. Bot. 56, 2239–2251 (2005). 15996984 10.1093/jxb/eri223

[b6] Wang J. L., Li T., Liu G. Y., Smith J. M. & Zhao Z. W. Unraveling the role of dark septate endophyte (DSE) colonizing maize (*Zea mays*) under cadmium stress: physiological, cytological and genic aspects. Sci Rep. 6, 22028 (2016). 26911444 10.1038/srep22028 PMC4766571

[b7] Sharma S. S., Dietz K. J. & Mimura T. Vacuolar compartmentalization as indispensable component of heavy metal detoxification in plants. Plant Cell Environ. 39, 1112–1126 (2016). 26729300 10.1111/pce.12706

[b8] Elobeid M., Gobel C., Feussner I. & Polle A. Cadmium interferes with auxin physiology and lignification in poplar. J. Exp. Bot. 63, 1413–1421 (2012). 22140243 10.1093/jxb/err384 PMC3276104

[b9] Qin W., Bazeille N., Henry E., Zhang B., Deprez E. & Xi X. G. Mechanistic insight into cadmium-induced inactivation of the Bloom protein. Sci Rep. 6, 26225 (2016). 27194376 10.1038/srep26225 PMC4872126

[b10] Schützendübel A. . Cadmium-induced changes in antioxidative systems, hydrogen peroxide content, and differentiation in scots pine roots. Plant Physiol. 127, 887–898 (2001). 11706171 10.1104/pp.010318 PMC129260

[b11] Stohs S. J. & Bagchi D. Oxidative mechanisms in the toxicity of metal ions. Free Radic. Biol. Med. 18, 321–336 (1995). 7744317 10.1016/0891-5849(94)00159-h

[b12] Semane B. . Cadmium responses in *Arabidopsis thaliana*: glutathione metabolism and antioxidative defence system. Physiol. Plant. 129, 519–528 (2007).

[b13] Migocka M. . Cucumber metal tolerance protein CsMTP9 is a plasma membrane H^+^-coupled antiporter involved in the Mn^2+^ and Cd^2+^ efflux from root cells. Plant J. 84, 1045–1058 (2015). 26485215 10.1111/tpj.13056

[b14] Greger M., Kabir A. H., Landberg T., Maity P. J. & Lindberg S. Silicate reduces cadmium uptake into cells of wheat. Environ. Pollut. 211, 90–97 (2016). 26745394 10.1016/j.envpol.2015.12.027

[b15] Vatamaniuk O. K., Mari S., Lang A., Chalasani S., Demkiv O. L. & Rea P. A. Phytochelatin synthase, a dipeptidyktransferase that undergoes multisite acylation with -glutamylcysteine during catalysis. J. Biol. Chem. 279, 22449–22460 (2004). 15004013 10.1074/jbc.M313142200

[b16] Cobbett C. & Goldsbrough P. Phytochelatins and metallothioneins: roles in heavy metal detoxification and homeostasis. Annu. Rev. Plant Biol. 53, 159–182 (2002). 12221971 10.1146/annurev.arplant.53.100301.135154

[b17] Tan B. H., Wong P. T. H. & Bian J. S. Hydrogen sulfide: a novel signaling molecule in the central nervous system. Neurochem. Int. 56, 3–10 (2010). 19703504 10.1016/j.neuint.2009.08.008

[b18] Papenbrock J., Riemenschneider A., Kamp A., SchulzVogt H. N. & Schmidt A. Characterization of cysteine-degrading and H_2_S-releasing enzymes of higher plants-from the field to the test tube and back. Plant Biol. 9, 582–588 (2007). 17853358 10.1055/s-2007-965424

[b19] Sirko A., Blaszczyk A. & Liszewska F. Overproduction of SAT and/or OASTL in transgenic plants: a survey of effects. J. Exp Bot. 55, 1881–1888 (2004). 15208350 10.1093/jxb/erh151

[b20] Kopriva S. Regulation of sulfate assimilation in Arabidopsis and beyond. Ann. Bot. 97, 479–495 (2006). 16464881 10.1093/aob/mcl006 PMC2803671

[b21] Alvarez C., Calo L., Romero L. C., Garcia I. & Gotor C. An O-acetylserine(thiol) lyase homolog with L-cysteine desulfhydrase activity regulates cysteine homeostasis in Arabidopsis. Plant Physiol. 152, 656–669 (2010). 19955263 10.1104/pp.109.147975 PMC2815857

[b22] Zhang L. & Liu J. Enhanced fatty acid accumulation in Isochrysis galbana by inhibition of the mitochondrial alternative oxidase pathway under nitrogen deprivation. Bioresour Technol. 211, 783–786 (2016). 27068057 10.1016/j.biortech.2016.03.164

[b23] Giraud E. . The absence of ALTERNATIVE OXIDASE1a in Arabidopsis results in acute sensitivity to combined light and drought stress. Plant Physiol. 147, 595–610 (2008). 18424626 10.1104/pp.107.115121 PMC2409015

[b24] Korshunov S., Imlay K. R. & Imlay J. A. The cytochrome bd oxidase of *Escherichia coli* prevents respiratory inhibition by endogenous and exogenous hydrogen sulfide. Mol Microbiol., doi: 10.1111/mmi. (2016). PMC4925259 26991114

[b25] Takahashi H., Kopriva S., Giordano M., Saito K. & Hell R. Sulfur Assimilation in Photosynthetic Organisms: Molecular Functions and Regulations of Transporters and Assimilatory Enzymes. Annu. Rev. Plant Biol. 62, 157–184 (2011). 21370978 10.1146/annurev-arplant-042110-103921

[b26] Christou A., Manganaris G. A., Papadopoulos I. & Fotopoulos V. Hydrogen sulfide induces systemic tolerance to salinity and non-ionic osmotic stress in strawberry plants through modification of reactive species biosynthesis and transcriptional regulation of multiple defence pathways. J. Exp. Bot. 647, 1953–1966 (2013). 10.1093/jxb/ert055 PMC3638822 23567865

[b27] Li J. S., Jia H. L., Wang J., Cao Q. & Wen Z. Hydrogen sulfide is involved in maintaining ion homeostasis via regulating plasma membrane Na^+^/H^+^ antiporter system in the hydrogen peroxide-dependent manner in salt-stress *Arabidopsis thaliana* root. Protoplasma 251, 899–912 (2014). 24318675 10.1007/s00709-013-0592-x

[b28] Li Z. G., Yang S. Z., Long W. B., Yang G. X. & Shen Z. Z. Hydrogen sulfide may be a novel downstream signal molecule in nitric oxide-induced heat tolerance of maize (*Zea mays L*.) seedlings. Plant Cell and Environ. 36, 1564–1572 (2013). 10.1111/pce.12092 23489239

[b29] Chen J. . Hydrogen sulfide alleviates aluminum toxicity in barley seedlings. Plant Soil 362, 301–318 (2013).

[b30] Li L., Wang Y. & Shen W. Roles of hydrogen sulfide and nitric oxide in the alleviation of cadmium-induced oxidative damage in alfalfa seedling roots. BioMetals. 25, 617–631 (2012). 22538639 10.1007/s10534-012-9551-9

[b31] Shi H., Ye T. & Chan Z. Nitric oxide-activated hydrogen sulfide is essential for cadmium stress response in bermudagrass (*Cynodon dactylon* (L). Pers.) Plant Physiol. Biochem. 74, 99–107 (2014). 24291156 10.1016/j.plaphy.2013.11.001

[b32] Sun J., Wang R., Zhang X., Yu Y., Zhao R., Li Z. & Chen S. Hydrogen sulfide alleviates cadmium toxicity through regulations of cadmium transport across the plasma and vacuolar membranes in Populus euphratica cells. Plant Physiol. Biochem. 65, 67–74 (2013). 23416498 10.1016/j.plaphy.2013.01.003

[b33] Zhang H. . Hydrogen sulfide promotes wheat seed germination and alleviates the oxidative damage against copper stress. J. Integr. Plant Biol. 50, 1518–1529 (2008). 19093970 10.1111/j.1744-7909.2008.00769.x

[b34] Jia H., Hu Y., Fan T. & Li J. Hydrogen sulfide modulates actin-dependent auxin transport viaregulating ABPs results in changing of root development in Arabidopsis. Sci. Rep. 5, 8251 (2015). 25652660 10.1038/srep08251 PMC4317700

[b35] Dooley F. D., Nair S. P. & Ward P. D. Increased growth and germination success in plants following hydrogen sulfide administration. Plos one 8, 62048–62052 (2013). 10.1371/journal.pone.0062048 PMC3629089 23614010

[b36] Zhang H. . Hydrogen Sulfide Promotes Root Organogenesis in *Ipomoea batatas, Salix matsudana* and *Glycine max*. J. Integr. Plant Biol. 51, 1086–1094 (2009). 20021556 10.1111/j.1744-7909.2009.00885.x

[b37] López-Martín M. C., Becana M., Romero L. C. & Gotor C. Knocking Out Cytosolic Cysteine Synthesis Compromises the Antioxidant Capacity of the Cytosol to Maintain Discrete Concentrations of Hydrogen Peroxide in Arabidopsis. Plant Physiol. 147, 562–572 (2008). 18441224 10.1104/pp.108.117408 PMC2409041

[b38] Wang J. & Vanlerberghe G. C. A lack of mitochondrial alternative oxidase compromises capacity to recover from severe drought stress. Physiol. Plant 149, 461–473 (2013). 23582049 10.1111/ppl.12059

[b39] Mostofa M. G., Rahman A., Ansary M. M., Watanabe A., Fujita M. & Tran L. S. Hydrogen sulfide modulates cadmium-induced physiological and biochemical responses to alleviate cadmium toxicity in rice. Sci. Rep. 5, 14078 (2015). 26361343 10.1038/srep14078 PMC4566128

[b40] Mostofa M. G., Hossain M. A., Fujita M. & Tran L. S. Physiological and biochemical mechanisms associated with trehaloseinduced copper-stress tolerance in rice. Sci. Rep. 5, 11433 (2015). 26073760 10.1038/srep11433 PMC4650698

[b41] Flores-Cáceres M. L., Hattab S., Boussetta H., Banni M. & Hernández L. E. Specific mechanisms of tolerance to copper and cadmium are compromised by a limited concentration of glutathione in alfalfa plants. Plant Sci. 233, 165–173 (2015). 25711824 10.1016/j.plantsci.2015.01.013

[b42] Li Y., Chen Y. Y., Yang S. G. & Tian W. M. Cloning and characterization of HbMT2a, a metallothionein gene from Hevea brasiliensis Muell. Arg differently responds to abiotic stress and heavy metals. Biochem. Biophys. Res. Commun. 461, 95–101 (2015). 25858315 10.1016/j.bbrc.2015.03.175

[b43] Fang H., Liu Z., Jin Z., Zhang L., Liu D. & Pei Y. An emphasis of hydrogen sulfide-cysteine cycle on enhancing the tolerance to chromium stress in Arabidopsis. Environ. Pollut. 213, 870–877 (2016). 27038574 10.1016/j.envpol.2016.03.035

[b44] Jian W. . Alternative oxidase pathway is involved in the exogenous SNP-elevated tolerance of *Medicago truncatula* to salt stress. J. Plant Physiol. 193, 79–87 (2016). 26962709 10.1016/j.jplph.2016.01.018

[b45] Sairam R. K. & Srivastava G. C. Changes in antioxidant activity in subcellular fraction of tolerant and susceptible wheat genotypes in response to long term salt stress. Plant Sci. 162, 897–904 (2002).

[b46] Freeman J. L. . Increased glutathione biosynthesis plays a role in nickel tolerance in Thlaspi nickel hyperaccumulators. Plant Cell 16, 2176–2191(2004). 15269333 10.1105/tpc.104.023036 PMC519206

[b47] Seth C. S. . Phytoextraction of toxic metals: a central role for glutathione. Plant Cell Environ. 35, 334–346 (2012). 21486307 10.1111/j.1365-3040.2011.02338.x

[b48] Zimeri A. M., Dhankher O. P., McCaig B. & Meagher B. M. The plant MT1 metallothioneins are stabilized by binding cadmium and are required for cadmium tolerance and accumulation. Plant Mol. Biol. 58, 839–855 (2005). 16240177 10.1007/s11103-005-8268-3

[b49] Heath R. L. & Packer L. Photoperoxidation in isolated chloroplasts: I. Kinetics and stochiometry of fatty acid peroxidation. Arch. Biochem. Biophys. 125, 189–198 (1968). 5655425 10.1016/0003-9861(68)90654-1

[b50] Nashef A. S., Osugam D. T. & Feeney R. E. Determination of hydrogen sulfide with 5,5β′-dithiobis-(2-nitrobenzoic acid), N-ethylmaleimide, and parachloromercuribenzoate. Anal. Biochem. 79, 394–405 (1977). 869184 10.1016/0003-2697(77)90413-4

[b51] Gaitonde M. K. A spectrophotometric method for the direct determination of cysteine in the presence of other naturally occurring amino acids. Biochem. J. 104, 627–633 (1967). 6048802 10.1042/bj1040627 PMC1270629

[b52] Mostofa M. G. & Fujita M. Salicylic acid alleviates copper toxicity in rice (*Oryza sativa L*.) seedlings by up-regulating antioxidative and glyoxalase systems. Ecotoxicology 22, 959–973 (2013). 23579392 10.1007/s10646-013-1073-x

[b53] Mostofa M. G., Yoshida N. & Fujita M. Spermidine pretreatment enhances heat tolerance in rice seedlings through modulating antioxidative and glyoxalase systems. Plant Growth Regul. 73, 31–44 (2014).

