# Supplementary material for: Hydrogen sulfide - cysteine cycle system enhances cadmium tolerance through alleviating cadmium-induced oxidative stress and ion toxicity in Arabidopsis roots
Source: Sci Rep. 2016 Dec 22;6:39702. doi: 10.1038/srep39702 (PMC5177925; doi:10.1038/srep39702)
Supplement: Supplementary Dataset 1 [file srep39702-s1.doc]

**Hydrogen sulﬁde and cysteine cycle system enhances cadmium tolerance through alleviating cadmium-induced oxidative stress and ion toxicity in Arabidopsis roots**

Honglei Jia^1,2^, Xiaofeng Wang^2^, Yanhua Dou^2^, Dan Liu^2^, Wantong Si^4^, Hao Fang^2,3^, Chen Zhao^2,3^, Shaolin Chen^2,3^, Jiejun Xi^5^, Jisheng Li^2,3,*^

^1^ School of Environmental Science and Engineering, Shaanxi University of Science & Technology, Xi'an, Shaanxi 710021, China

^2^ College of Life Sciences, Northwest A&F University, Yangling, Shaanxi 712100, China

^3^ Biomass Energy Center for Arid and Semi-arid Lands, Northwest A&F University, Shaanxi 712100, China

^4^ Inner Mongolia Key Laboratory of Biomass-Energy Conversion, Inner Mongolia University of Science and Technology. Baotou, Neimenggu, 014010, China

^5^ Department of Grassland Science, College of Animal Science and Technology, Northwest A&F University, Shaanxi 712100, China

*Author to whom correspondence should be addressed: Jisheng Li

Fax: +86 029 87092262, E-mail: lijsh2011@163.com

**Table S1. List of all genes for qRT-PCR in the manuscript.**

| **Gene** | **Accession number** | **Primer pairs** |
| --- | --- | --- |
| *AOX1A* | AT3G22370 | ACTAGAGCTCCGACGATTGG |
|  |  | ATACGTTTCCCATGGCCTGA |
| *AOX1C* | AT3G27620 | TGGTACGAACGAGCTCTTGT |
|  |  | GCCTCCAGTAATCAACAGCG |
| *AOX2* | AT5G64210 | GATCGGATTGGCCTTGGAAC |
|  |  | TGACAATCCGGTAAGCGACT |
| *LCD* | AT3G62130 | CGTGTGGGAGGTTCTACTGT |
|  |  | CACACCTTCCTCTCTGCAGA |
| *DES1* | AT5G28030 | ATTGTGGATGGTTGTGTGGC |
|  |  | CCCGTTGCCTCAATCAATGT |
| *D-CDES* | AT1G48420 | AGGGAAATTGAGGAGCAGCT |
|  |  | AGCGTGAAGTCCATCCAGAA |
| *SAT1* | At1g55920 | CTTTTAGACCATGCGACGGG |
|  |  | ACTCCCAGCTCCAATCAACA |
| *SAT3* | AT3G13110 | ACCGGGATTTTGCTAGACCA |
|  |  | GTCTTTCAACACCACCGACC |
| *SAT5* | AT5G56760 | AAACAGCGGTGATTGGGAAC |
|  |  | ATCAGCACAACAGAACCAGC |
| *OASA1* | AT4G14880 | GATGTGTTGGTCGTGTTGCT |
|  |  | ACCCAACTCCAGTGTTTCCA |
| *OASB* | AT2G43750 | AACCCGGACCTCACAAGATT |
|  |  | CAACGGCTATGAGTTTCCCG |
| *OASC* | At3g59760 | ATGACTGGAGCGGTTCAGAA |
|  |  | CCACCAGTTCCAATTCCTGC |
| *MT1A* | AT1G07600 | TAACTGTGGATGTGGCTCCT |
|  |  | CAGCTGCAGTTTGATCCACA |
| *MT1B* | AT5G56795 | ATTGTGGATGTGGCTCCTCC |
|  |  | GTTACAGTTTGACCCACAGCT |
| *MT2A* | AT3G09390 | GGAAACTGCGGATGTGGATC |
|  |  | CCCTGAAGCCTCGTACTGAT |
| *PCS1* | AT5G44070 | GAAAGTGGAAAGGGCCTTGG |
|  |  | GTGCTCTGATTTGTGCGGAA |
| *PCS2* | AT1G03980 | GAAAGTGGAAAGGGCCTTGG |
|  |  | GTGCTCTGATTTGTGCGGAA |
